# Editorial: Statistical methods for genome-wide association studies (GWAS) and transcriptome-wide association studies (TWAS) and their applications

**DOI:** 10.3389/fgene.2023.1287673

**Published:** 2023-09-11

**Authors:** Mengting Shao, Zilong Zhang, Huiyan Sun, Jingni He, Juexin Wang, Qingrun Zhang, Chen Cao

**Affiliations:** ^1^ School of Biomedical Engineering and Informatics, Nanjing Medical University, Nanjing, China; ^2^ School of Computer Science and Technology, Hainan University, Haikou, China; ^3^ School of Artificial Intelligence, Jilin University, Changchun, China; ^4^ Department of Biochemistry and Molecular Biology, University of Calgary, Calgary, AB, Canada; ^5^ Department of Biohealth Informatics, Indiana University Purdue University Indianapolis, Indianapolis, IN, United States; ^6^ Department of Mathematics and Statistics, University of Calgary, Calgary, AB, Canada

**Keywords:** genome-wide association studies, transcriptome-wide association studies, linear mixed models, bayesian models, pathway analysis, statistical methodology, meta-analysis

## Introduction

The Genome-Wide Association Studies (GWAS) has proven highly successful in identifying millions of risk loci associated with various diseases in the past 15 years (Klein et al., 2005). With the rapid accumulation of GWAS summary-level data, biologists now have expanded opportunities to uncover new disease-associated variants and gain insights into the mechanisms underlying complex human diseases (Michailidou et al., 2017; Sud et al., 2017; Zhang et al., 2020). While GWAS is a powerful tool, it faces challenges in pinpointing candidate disease risk genes. For instance, many disease-associated variants reside in non-coding regions, complicating the identification of their regulatory genes and underlying mechanisms due to the likely cell-type, context, and disease-specific effects of non-coding causal variants (Kossinna et al., 2022). On the other hand, GWAS finds it hard to distinguish between causal variant signals and significance signals in high Linkage Disequilibrium (LD) regions, leading to poor interpretation of GWAS signals (Christoforou et al., 2012; Cao et al., 2021a).

In response to these challenges, several post-GWAS methods have emerged, including Transcriptome-Wide Association Studies (TWAS) (Gamazon et al., 2015; Cao et al., 2021b; Cao et al., 2022; He et al., 2022), Proteome-Wide Association Study (PWAS) (Brandes et al., 2020), and Summary Data-Based Mendelian Randomization (SMR) (Zhu et al., 2016). These methods serve as potent tools for discovering candidate disease risk genes, offering benefits such as enhanced statistical power, improved interpretability, and reduced computational costs. In recent years, numerous studies have adopted GWAS, TWAS, and SMR to delve into the intricate biological mechanisms of diseases (Baca et al., 2022).

## Causal variants in multiple traits

Untangling causal signals from mere associations in GWAS presents a big challenge. Techniques such as fine mapping, Mendelian randomization, and TWAS have risen to address this challenge, facilitating the translation of GWAS findings into a functional understanding of associated traits. In this Research Topic of Frontiers in Genetics, six research articles demonstrate the efficacy of these techniques. For instance, Lu et al. utilized meticulous fine mapping to identify the rs7175517 variant as related to Body Mass Index (BMI) across diverse populations, offering fresh insights into the global obesity epidemic. In another study, Chen et al. used blood proteins as traits in GWAS, employing a two-sample Mendelian randomization analysis to identify causal proteins linked to sarcopenia-related traits. This not only identified potential therapeutic targets but also shed light on underlying genetic factors. In a separate investigation, Lu et al. focused on celiac disease, a comprehensive strategy involving TWAS and chemical-gene interaction analyses unveiled celiac disease-related genes and chemicals, providing valuable insights at both the genetics and environmental levels.

## Various tools in genome-wide association studies

Another critical aspect of identifying disease-associated genes involves prioritizing trait-specific tissues, which may lead to differences in gene expression and variant regulation. To address this, Ghaffar and Nyholt developed a method called genome-wide imputed differential expression enrichment (GIDEE). GIDEE prioritizes pathogenic tissues by analyzing the enrichment of differentially expressed genes in each tissue. Additionally, the relationship between diseases plays a key role in identifying variants shared across multiple traits or diseases. To tackle this challenge, graph-GPA 2.0 (GGPA 2.0) was proposed by Deng et al. It integrates GWAS datasets of multiple diseases and utilizes functional annotations within a unified framework, successfully detecting pleiotropy between bipolar disorder and schizophrenia. Furthermore, a Visual SNP interpretation tool named SNPMap was proposed by Liu et al. to illustrate semantic relations between SNPs and traits, significance, and SNP-related information. This tool aids researchers in better understanding the link between genetic variation and disease risk.

## Conclusion

Together, these articles reveal the significant potential of identifying disease susceptibility genes, understanding disease mechanisms, and discovering drug targets using GWAS and post-GWAS tools. They provide valuable knowledge resources for future medical research and clinical applications.
